# Alterações Hematológicas durante um Período de Sete Dias de Internação em Pacientes com Infarto Agudo do Miocárdio

**DOI:** 10.36660/abc.20230045

**Published:** 2023-10-26

**Authors:** Cyntia Maria de Holanda Martins, José Gildo de Moura Monteiro, Dilênia de Oliveira Cipriano Torres, Dario Celestino Sobral, Maria Clara Santos Morais, Ulisses Ramos Montarroyos, Izadora Karina da Silva, Ana Célia Oliveira dos Santos

**Affiliations:** 1 Instituto de Ciências Biológicas Universidade de Pernambuco Recife PE Brasil Instituto de Ciências Biológicas - Universidade de Pernambuco (UPE), Recife, PE – Brasil; 2 Pronto Socorro Cardiológico de Pernambuco UPE Recife PE Brasil Pronto Socorro Cardiológico de Pernambuco (PROCAPE-UPE), Recife, PE – Brasil; 3 Faculdade de Ciências Médicas Universidade de Pernambuco Recife PE Brasil Faculdade de Ciências Médicas - Universidade de Pernambuco (FCM-UPPE), Recife, PE – Brasil

**Keywords:** Infarto do Miocárdio/mortalidade, Anisocitose, Amplitude de Distribuição dos Glóbulos Vermelhos, Placa Aterosclerótica/fisiopatologia, Prognóstico

## Abstract

**Fundamento:**

O infarto agudo do miocárdio é uma das principais causas de mortalidade em todo o mundo e a formação de placa aterosclerótica é o principal mecanismo fisiopatológico, que resulta em inflamação crônica e induz a maturação eritrocitária, podendo causar aumento no índice de amplitude de distribuição dos glóbulos vermelhos (RDW).

**Objetivo:**

Avaliar o papel do índice de anisocitose em pacientes com infarto agudo do miocárdio em ambos os tipos de infarto como preditor de gravidade.

**Métodos:**

Os pacientes foram incluídos no estudo de acordo com os critérios de inclusão e exclusão, seguindo a rotina hospitalar baseada na história clínica e laboratorial. As análises estatísticas foram realizadas de acordo com cada variável. Chegou-se a todas as conclusões considerando o nível de significância de 5%.

**Resultados:**

Durante o período de acompanhamento, nos 349 pacientes analisados, a taxa de mortalidade esteve associada às variáveis RDW (CV) e RDW (SD). Nos pacientes que foram a óbito, notou-se aumento, conforme demonstrado no modelo multivariado, nos efeitos de um infarto agudo do miocárdio com supradesnivelamento do segmento ST e RDW, ajustado para fatores de confusão (valor-p = 0,03 e 0,04). Em contrapartida, o número total de eritrócitos (valor-p = 0,00) e hemoglobina (valor-p = 0,03) apresentou diminuição durante a internação de pacientes graves.

**Conclusão:**

O índice de anisocitose foi fator preditivo de mortalidade e pode ser utilizado como indicador de pior prognóstico em pacientes com infarto agudo do miocárdio.

**Figura Central:**
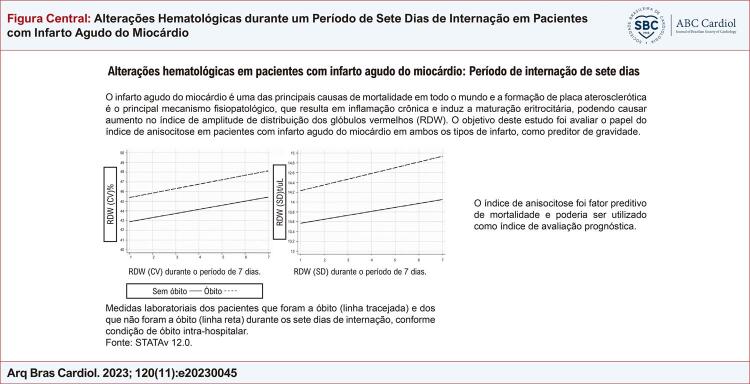
: Alterações Hematológicas durante um Período de Sete Dias de Internação em Pacientes com Infarto Agudo do Miocárdio

## Introdução

O infarto agudo do miocárdio (IAM) é um importante problema de saúde em todo o mundo, causando morbidade e mortalidade.^1^ Os primeiros sintomas do IAM manifestam-se nas primeiras horas e, sem assistência médica, muitas vezes resultam em mortalidade.^2^ O infarto pode ser dividido em seis categorias: infarto por aterotrombose coronariana (tipo 1); infarto devido à incompatibilidade entre oferta e demanda do miocárdio, não devido a aterotrombose coronariana (tipo 2); infarto com morte súbita sem oportunidade de comprovação bioquímica ou eletrocardiográfica (tipo 3); infarto relacionado à intervenção coronariana percutânea (angioplastia coronariana) (tipo 4a); infarto relacionado à trombose de stent coronariano (4b); e infarto relacionado à cirurgia de revascularização miocárdica (tipo 5). Trata-se de uma doença multifatorial que, em muitos casos, dificulta o diagnóstico e o tratamento. Consequentemente, torna-se importante o uso de biomarcadores que forneçam informações sobre a gravidade do evento.^3 , 4^A amplitude de distribuição dos glóbulos vermelhos (RDW), um biomarcador também conhecido como índice de anisocitose, é expressa pela quantificação da variabilidade de tamanho dos glóbulos vermelhos e, juntamente com as alterações hematológicas dos eritrócitos, hemoglobina e hematócrito, é analisada rotineiramente em emergências cardíacas.^5^ O RDW pode ser relatado, dependendo da análise estatística, tanto como o coeficiente de variação (RDW-CV) e/ou como desvio padrão, (RDW-SD).

Estudos demonstraram uma capacidade preditiva do aumento do RDW, refletindo diversas complicações durante e após a ocorrência do infarto.^6 , 7^ Em relação à inflamação crônica, aspectos multifatoriais como idade, sexo, genética, hormônios, medicamentos e dieta podem modular a biologia e a fisiologia dos eritrócitos. Assim, esses aspectos podem ser considerados na patogênese do infarto, no qual a liberação de vias em cascata, como certas citocinas, por exemplo, pode afetar a regulação da medula óssea com a consequente maturação dos eritrócitos, o que afeta a taxa de eritropoiese e o tamanho do eritrócitos circulantes o que, por sua vez, influenciam no aumento do RDW.^8 , 9^ A formação de placas ateroscleróticas, principal causa do infarto, é resultado da inflamação crônica que induz a maturação dos eritrócitos, causando um aumento no índice RDW.^10 - 12^ Portanto, se faz necessário estudar e monitorar biomarcadores a fim de auxiliar nos resultados clínicos e, assim, determinar um melhor prognóstico imunológico e hematológico associado a doenças multifatoriais.^13^ A utilização de alterações hematológicas como biomarcadores tem suas vantagens. Essas alterações são medidas simples e baratas, e podem ser avaliadas rotineiramente além de auxiliar na estratificação de pacientes com doenças cardiovasculares na prática clínica. Também apresentam vantagem especial em relação à mortalidade em pacientes com infarto agudo do miocárdio. Assim, o presente artigo tem como objetivo avaliar as alterações do RDW e compará-la com o prognóstico de pacientes com infarto agudo do miocárdio durante sete dias de internação em um hospital de emergência.

## Métodos

### Desenho do estudo

Trata-se de um estudo prospectivo, observacional, com dados coletados de janeiro a setembro de 2018, com pacientes infartados, em um hospital universitário referência em cardiologia. Os pacientes foram selecionados e divididos em dois grupos, de acordo com o tipo de infarto: aqueles com supradesnivelamento do segmento ST (IAMCSST) e aqueles sem supradesnivelamento do segmento ST (sem IAMCSST). As avaliações clínicas, eletrocardiográficas e laboratoriais foram realizadas pelo médico plantonista e revisadas pelos pesquisadores. O acompanhamento e tratamento dos pacientes seguiram os protocolos institucionais. Informações gerais como idade, sexo, presença de comorbidades e desfechos clínicos foram obtidas dos prontuários dos pacientes. Os pacientes foram acompanhados por um período de sete dias e os desfechos foram registrados exatamente de acordo com as informações do prontuário, pela equipe médica responsável.

### População do estudo

Foram incluídos pacientes internados no hospital com IAM durante o período do estudo. Foram excluídos do estudo pacientes menores de 18 anos, gestantes, portadores de doenças hematológicas ou oncológicas, que fizeram uso prévio de corticoide ou quimioterapia e pacientes novamente internados após alta hospitalar. Todos os pacientes assinaram um termo de consentimento livre e esclarecido para participar do estudo.

### Definição dos termos e variáveis do estudo

Foram realizados exames laboratoriais com amostras de sangue coletadas diariamente por punção venosa até as 9h. Foram respeitados critérios rigorosos de controle de qualidade. As análises laboratoriais foram realizadas com o sistema Sysmex XE-2100 (Sysmex Europe GmbH, Norderstedt, Alemanha). Os pontos de corte das variáveis hematológicas analisadas foram: Hematócrito (%): Mulheres (35 - 47), Homens (40 - 54); Glóbulos vermelhos (milhões/mm ^3^ ): Mulheres (4,0 - 5,6), Homens (4,5 - 6,5); Hemoglobina (g/100 ml): Mulheres (12 - 16,5), Homens (13,5 - 18); Plaquetas: 140.000 a 450.000 (µl) para ambos os sexos; RDW-CV 12 em 14,4%; e RDW-SD 38,6 a 49,1fL, para ambos os sexos.

Possíveis fatores de risco associados ao IAM, como características demográficas (idade, sexo), hipertensão arterial sistêmica (pressão arterial ≥ 140 x 90 mmHg), diabetes mellitus (glicemia plasmática acima de 126 mg/dL) e sedentarismo (prática regular de exercício físico ou não), foram ajustados pelo modelo estatístico.

Durante as primeiras vinte e quatro horas de internação, os pacientes também foram classificados como infectados (sepse) e não infectados, seguindo os critérios da síndrome da resposta inflamatória sistêmica, além de foco infeccioso documentado ou presumido (uso de antibióticos).

### Análise estatística

As variáveis categóricas foram apresentadas por meio de frequências absolutas e relativas e comparadas por meio do teste Qui-Quadrado (teste χ ^2^ ). Os dados quantitativos com distribuição normal foram apresentados por meio de média e desvio padrão e as comparações entre os grupos foram realizadas pelo teste t de Student não pareado quanto às características dos pacientes com infarto agudo do miocárdio relacionadas a óbito e relacionadas a supradesnivelamento do segmento ST e sem supradesnivelamento do segmento ST. O teste aplicado para comprovar a distribuição normal foi o teste de Kolmogorov-Smirnov. Um modelo GGE (Equações de Estimativas Generalizadas) para medidas repetidas foi aplicado para estimar o efeito ao longo do tempo de cada medida laboratorial relacionada ao óbito durante a hospitalização. Foi realizado um modelo multivariado do efeito do supradesnivelamento do segmento ST e do RDW na mortalidade, ajustado para fatores de confusão. O nível de significância estatística adotado foi de 5%. Após a transferência dos dados compilados em Word e Excel para o programa STATA, todo o banco de dados passou por um processo de verificação de três pontos para detectar possíveis inconsistências e erros de digitação antes da elaboração dos relatórios estatísticos.

### Declaração de ética

O presente estudo faz parte da linha de projetos (Biomarcadores em Pesquisa Clínica) aprovado pelo Comitê de Ética do Complexo Hospitalar HUOC/PROCAPE da Universidade de Pernambuco, sob CAAE: 51802115.7.0000.5192 (Plataforma Brasil). Todos os procedimentos deste estudo estão de acordo com a Declaração de Helsinque de 1975, atualizada em 2013. Foi obtido consentimento livre e esclarecido de todos os participantes incluídos no estudo.

## Resultados

**Figura 1:**
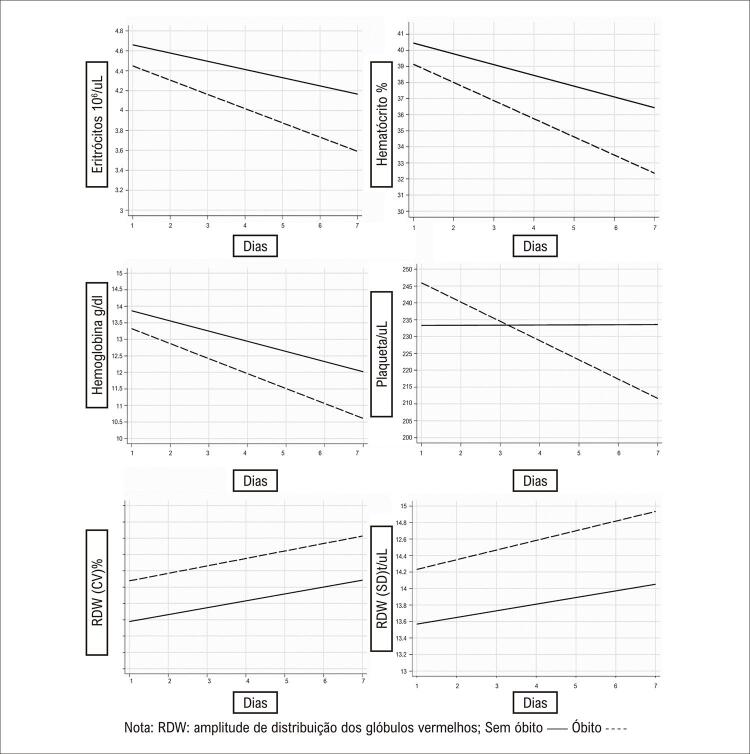
– Medidas laboratoriais dos pacientes que foram a óbito (linha tracejada) e dos que não foram a óbito (linha reta) durante os sete dias de internação, conforme condição de óbito intra-hospitalar.

Houve alterações nos dados hematológicos dos pacientes infartados durante os sete dias de internação, com aumento do RDW de acordo com o tempo de internação, tanto para os pacientes que foram a óbito quanto para os que não foram a óbito, sem associação com o tipo de ataque cardíaco ( Figura Central ). Os níveis de eritrócitos, hemoglobina e hematócrito diminuíram ao longo do período de internação, independentemente do tipo de infarto ( Figura 1 ).

O presente estudo incluiu um total de 349 participantes da pesquisa. Houve predomínio de infarto agudo do miocárdio com supradesnivelamento do segmento ST (IAMCSST) (70,4%). A Tabela 1 apresenta as características dos pacientes com IAMCSST e sem IAMCSST. Somando-se os níveis à tabela geral, os níveis de RDW, tanto RDW (CV) quanto RDW (SD), analisados durante os primeiros sete dias de internação, foram bastante semelhantes entre os dois tipos de IAM.

**Tabela 1:** – Características dos pacientes com infarto agudo do miocárdio relacionadas a supradesnivelamento do segmento ST e sem supradesnivelamento do segmento ST

Características	Total (n = 349)	Non –ST (n = 103)	ST (n = 246)	Valor-p
**Idade (média ± SD)**	64,4 ± 12,7	67,9 ± 13,36	63,2 ± 12,1	0,008
**Sexo: Masculino**	222 (63,6%)	47 (45,6%)	80 (32,5%)	0,020
**Fatores de risco**				
**HAS**				
Sim	254 (72,7%)	88 (85,4%)	166 (67,4%)	0,001
Não	95 (27,3%)	15 (14,5%)	80 (32,5%)	
**DM**				
Sim	128 (36,6%)	39 (37,4%)	89 (36,1%)	0,760
Não	221 (63,2%)	64 (62,1%)	157 (63,8%)	
**Dislipidemia**				
Sim	133 (38,1%)	50 (48,5%)	83 (33,7%)	0,009
Não	216 (61,8%)	53 (51,4%)	163 (66,2%)	
**Desfechos**				
**Cirurgia**				
Sim	22 (6,9%)	13 (12,6%)	11 (4,4%)	0,006
Não	325 (96,1%)	90 (87,3%)	235 (95,5%)	
**Angioplastia**				
Sim	174 (49,8%)	22 (21,3%)	152 (61,7%)	0,000
Não	175 (50,14%)	81 (78,6%)	94 (38,2%)	
**Óbito**				
Não	305 (87,3%)	94 (91,6%)	211 (85,7%)	0,159
Sim	44 (12,61%)	9 (8,74%)	35 (14,23%)	
* RDW % (CV)	13,8 (± 2,25)	13,5 (± 0,3)	13,2 (± 1,3)	0,817
* RDW fL (SD)	44,1 (± 10,2)	43,3 (± 4,3)	43,5 (± 4,7)	
**Sepse**				
Sim	24 (6,8%)	8 (7,7%)	16 (6,5%)	0,671
Não	325 (93,1%)	95 (92,2%)	130 (93,5%)	

HAS: hipertensão arterial sistêmica; DM: Diabetes mellitus; RDW: amplitude de distribuição dos glóbulos vermelhos* média e desvio padrão do RDW (coeficiente de variação) e RDW (desvio padrão) obtidos durante os primeiros sete dias de internação. ST: Infarto do miocárdio com supradesnivelamento do segmento ST.

Foram encontradas diferenças significativas nas variáveis idade, ocorrência de hipertensão arterial sistêmica, sedentarismo, procedimentos cirúrgicos e angioplastia, conforme descrito na Tabela 1 . A análise estatística foi realizada comparando as características gerais com a mortalidade dos pacientes ( Tabela 2 ). Houve uma taxa de mortalidade de 44 (12,6%). A maioria dos que faleceram eram pacientes do sexo masculino com supradesnivelamento do segmento ST (14,23%), com idade média de 63 anos. A alta hospitalar apresentada na Tabela 1 foi o desfecho mais prevalente (87,3%) e predominou em pacientes sem supradesnivelamento do segmento ST (91,6%).

**Tabela 2:** – Características dos pacientes com infarto agudo do miocárdio relacionadas a morte

Características	Nenhuma morte (n = 305)	Óbito (n = 44)	Valor-p
**Idade (média ± SD)**	63,5 ± 12,7	72 ± 10,1	0,008
**Sexo: Masculino**	194 (63,6%)	28 (63,6%)	0,997
**Comorbidades**			
**HAS**			
Sim	217 (71,1%)	37 (84,0%)	0,070
Não	88 (28,8%)	7 (15,9%)	
**DM**			
Sim	107 (35,0%)	21 (47,7%)	0,104
Não	198 (64,9%)	23 (52,2%)	
**Dislipidemia**			
Sim	118 (38,6%)	15 (34,0%)	0,557
Não	187 (61,3%)	29 (65,9%)	
**Resultado**			
**Cirurgia**			
Sim	22 (7,2%)	2 (4,6%)	0,513
Não	283 (92,7%)	42 (95,4%)	
**Angioplastia**			
Sim	152 (49,8%)	22 (50,0%)	0,984
Não	153 (50,2%)	22 (50,0%)	
**Sepse**			
Sim	12 (3,9%)	12 (27,2%)	0,000
Não	293 (96,0%)	32 (72,7%)	

HAS: hipertensão arterial sistêmica; DM: Diabetes mellitus.

Os dados laboratoriais foram obtidos ao longo dos sete dias por meio da diferença média nas medidas laboratoriais entre os pacientes com supradesnivelamento do segmento ST ( Tabela 3 ). Houve diminuição dos níveis de hematócrito e hemoglobina dos eritrócitos. A contagem total de eritrócitos apresentou diferença estatisticamente significativa nesta avaliação.

**Tabela 3:** – Estimativa de dados laboratoriais ao longo do tempo e diferença média nas medidas laboratoriais entre pacientes com supradesnivelamento do segmento ST. Modelo de regressão logística multivariada e valor-p

Medidas laboratoriais	Efeito ao longo do tempo	Avaliação ST	Valor-p	Avaliação ST ajustada	CI (95%)	Valor-p
Eritrócitos ^a^	-0,078	-0,022	0,751	-0,143	-0,28 a -0,01	0,041
Hemoglobina ^a^	-0,280	0,092	0,679	-0,369	-0,81 a 0,08	0,107
Hematócrito ^a^	-0,629	0,266	0,644	-0,762	-2,06 a 0,61	0,287
Plaquetas ^a^	-0,110	6,57	0,404	5,66	-11.1 a 22.4	0,507
RDW (SD) ^a^	0,464	-0,127	0,879	0,005	-0.03 a 0.04	0,756
RDW (CV) ^a^	0,080	-0,302	0,102	-0,025	-0,06 a 0,07	0,123

RDW: amplitude de distribuição dos glóbulos vermelhos. ^a^ Ajustado por sexo, idade, hipertensão, dislipidemia, cirurgia e angioplastia.

O nível da contagem total de eritrócitos variou em -0,078, com diminuição dos níveis, causando, consequentemente, efeito negativo, de acordo com o tipo de infarto, ao longo do tempo. Quando ajustada pelos fatores sexo, idade, hipertensão, sedentarismo, cirurgia e angioplastia, a anisocitose teve efeito positivo ao longo do tempo em pacientes com e sem supradesnivelamento do segmento ST. Foi observada significância estatística para RDW (CV) e RDW (SD), bem como para contagem total de eritrócitos e concentração de hemoglobina, que foram (p < 0,006) e (p < 0,032), respectivamente ( Tabela 4 ).

**Tabela 4:** – Estimativa dos dados laboratoriais ao longo do tempo e diferença média das medidas laboratoriais ao longo do tempo entre pacientes que faleceram e não faleceram durante a internação. Modelo de regressão logística multivariada e valor-p

Medidas laboratoriais	Efeito ao longo do tempo	Suprades-nivelamento do segmento ST	Valor-p	Suprades-nivelamento do segmento ST ajustado	IC (95%)	Valor-p
Eritrócitos ^a^	-0,077	-0,362	< 0,001	-0,243	-0,42 a -0,07	0,00
Hemoglobina ^a^	-0,283	-0,922	0,002	-0,619	-1,18 a -0,05	0,03
Hematócrito ^a^	-0,620	-2,56	0,001	-0,730	-2,30 a 0,84	0,36
Plaquetas ^a^	-0,09	-7,040	0,404	-7,160	-28,8 a 14,5	0,51
RDW (SD) ^a^	0,431	2,619	0,013	2,277	0,14 a 4,41	0,03
RDW (CV) ^a^	0,073	0,76	0,001	0,531	0,02 a 1,04	0,04

RDW: amplitude de distribuição dos glóbulos vermelhos. ^a^ Ajustado para idade, hipertensão e septicemia.

## Discussão

Os dados do presente estudo avaliaram alterações no índice de anisocitose (RDW) em pacientes com IAM nos dois tipos de infartos, aqueles com IAMCSST e aqueles sem IAMCSST. Os índices hematológicos medidos rotineiramente nos exames laboratoriais de emergência avaliados neste estudo demonstraram que a presença de anisocitose em pacientes infartados está associada a maior mortalidade no infarto agudo do miocárdio, especialmente naqueles com supradesnivelamento do segmento ST (IAMCSST). Dados de hemograma completo são rotina em emergências cardíacas e podem contribuir para o atendimento médico prestado por meio de informações de fácil acesso.

Durante a última década, tem havido um interesse crescente em estudos voltados ao uso do RDW como um novo marcador para o prognóstico e gravidade das doenças cardiovasculares.^8 , 10 , 12^ Ye et al.,^14^ analisaram a utilidade do RDW em pacientes com doença arterial periférica e relataram associação positiva entre aumento do RDW e mortalidade.^14^ O presente estudo descreveu as condições dos pacientes, no momento da hospitalização e ao longo dos sete dias de internação, de acordo com o acompanhamento hospitalar de rotina, incluindo a avaliação dos escores de risco utilizados pela equipe de cardiologia. A maior parte da população avaliada era composta por idosos, com idade média de 64 anos ( Tabela 1 ), condição que está associada a doenças cardiovasculares e à gravidade da doença.^15 - 17^

Nossos achados vão ao encontro do estudo de Arbel et al.,^18^ que relatou que em pacientes com supradesnivelamento do segmento ST, o índice de anisocitose esteve associado a maiores taxas de mortalidade, com influência sobre os efeitos fisiopatológicos do sedentarismo e, consequentemente, diagnóstico e tratamento de pacientes com IAM. Ao comparar os níveis de RDW em pacientes com e sem supradesnivelamento do segmento ST, não foi observada diferença estatística entre as duas condições. No entanto, observou-se um aumento progressivo em relação aos níveis de referência.

O estudo de Vaya et al.,^19^ com 199 pacientes infartados, concluiu que níveis de RDW (CV) acima de 14% estavam diretamente associados a um aumento de seis vezes nos eventos cardiovasculares, mesmo quando ajustado para anemia. A ingestão de água pode predispor os indivíduos a um maior risco futuro de eventos cardiovasculares adversos, uma vez que há evidências de que a hipohidratação aguda prejudica a função vascular e a regulação da pressão arterial.^20^ Neste estudo, as concentrações eletrolíticas nos pacientes foram controladas.

Em termos gerais, a análise multivariada apresentou aumento da anisocitose quando comparado à evolução durante os sete dias de internação entre os pacientes que foram e não foram a óbito. A determinação do RDW pode ser capaz de identificar alterações reológicas nas propriedades dos glóbulos vermelhos, influenciando, por exemplo, a agregação das células e consequentemente a viscosidade e a taxa de fluxo sanguíneo, gerando consequências adversas, dependendo do paciente.^21^

Níveis elevados de anisocitose indicam produção de células imaturas pela medula óssea, afetando sua atividade.^22^ O aumento da viscosidade sanguínea está relacionado a alterações no índice de anisocitose como efeito do aumento do processo de agregação e fragmentação celular, conforme relatado por Neuman et al., que identificaram diferença significativa na sobrevida de pacientes com angina instável que apresentavam níveis elevados de agregação de glóbulos vermelhos durante a internação. Isso demonstra que o índice de anisocitose pode estar relacionado à taxa de fluxo sanguíneo e à capacidade de microoclusão, influenciando diretamente pacientes com infarto agudo do miocárdio.^23 - 25^

O presente estudo demonstrou que o RDW é estatisticamente significativa quando analisada em conjunto com medidas hematológicas (eritrócitos, hemoglobina, hematócrito e plaquetas) ao longo do tempo, quando utilizado o cálculo de regressão que considera a variável mortalidade.

A análise multivariada apresentou um padrão de diminuição das variáveis hematológicas em contraste com o aumento progressivo do RDW-CV e RDW-SD, de acordo com os dias de internação. Esse achado pode ter ocorrido porque uma alteração no padrão eritrocitário em condições hipóxicas altera diretamente funções essenciais e aumenta o processo inflamatório e o estresse oxidativo. Além disso, pode interferir na formação de diversos complexos, cruciais para a homeostase como um todo, afetando a eritropoiese, o que induz à maturação eritrocitária deficiente, parcialmente no RDW.^26 - 28^

Os pacientes avaliados neste estudo apresentaram níveis de hemoglobina abaixo da normalidade. Nos casos de anemia, a alteração no RDW se deve à insuficiente maturação eritrocitária, na tentativa de suprir o suprimento de oxigênio, já que a presença de anisocitose é uma população heterogênea de eritrócitos causada por distúrbios na fase de hemoglobinização.^29^ Essa redução é explicada como consequência de obstruções na artéria coronária, que consiste na perda do suprimento sanguíneo e resulta em isquemia e morte celular em toda a região irrigada pela artéria.^30^ No entanto, isso depende da gravidade e da duração da privação de fluxo.^31^

Foi observada associação inversa de anisocitose com níveis de hemoglobina. Esses dados estão relacionados ao fato de que a diminuição da hemoglobina é um indicador de baixa oxigenação devido a patologias como o infarto, o que pode levar a diversas variáveis hematológicas adversas, por exemplo, um aumento do RDW.^32^

Procedimentos de angioplastia coronariana foram realizados em 49,8% dos pacientes. Entretanto, esse procedimento foi considerado mais necessário em pacientes com supradesnivelamento do segmento ST (p < 0,000). A diminuição do fluxo pós-angioplastia já foi associada anteriormente ao aumento dos níveis de RDW, e está sempre relacionada a um pior prognóstico.^33^

As angioplastias, assim como outros procedimentos cirúrgicos, também afetam diretamente o perfil do paciente, bem como o fator tecidual no papel da doença, tornando o paciente imunologicamente mais frágil, uma vez que o histórico de septicemia está diretamente associado ao maior número de óbitos e esses fatores estão intrinsecamente relacionados.^34^

O presente estudo é limitado porque os pacientes foram selecionados em um único centro; portanto, os resultados podem refletir a prática local. Para diminuir essa limitação, aumentamos o tamanho da amostra e utilizamos protocolo padronizado e pré-determinado para minimizar possíveis vieses. As informações sobre comorbidades e condições de tratamento foram extraídas de prontuários e da rotina do pronto-socorro cardiológico, o que pode apresentar possíveis vieses. Outra limitação importante é que o estudo não permitiu ajustar os resultados para outros indicadores de gravidade, como insuficiência renal, uso de anticoagulantes e sangramento. Essas variáveis estão sendo objeto de novas pesquisas do grupo.

## Conclusões

O papel do índice de anisocitose em pacientes com IAM em ambos os tipos de infarto foi analisado, demonstrando fator preditivo de gravidade durante sete dias de internação. O estudo procurou proporcionar uma melhor compreensão do perfil do índice RDW, juntamente com o perfil hematológico para predição de desfechos em pacientes infartados.
