# Supplementary material for: Alterações Hematológicas durante um Período de Sete Dias de Internação em Pacientes com Infarto Agudo do Miocárdio
Source: Arq Bras Cardiol. 2023 Oct 26;120(11):e20230045. [Article in Portuguese] doi: 10.36660/abc.20230045 (PMC12080720; doi:10.36660/abc.20230045)
Supplement: Translation

# Translation: Hematological Changes during Seven Days of Hospitalization in Patients with Acute Myocardial Infarction

**Authors:** Cyntia Maria de Holanda Martins, José Gildo de Moura Monteiro, Dilênia de Oliveira Cipriano Torres, Dario Celestino Sobral, Maria Clara Santos Morais, Ulisses Ramos Montarroyos, Izadora Karina da Silva, Ana Célia Oliveira dos Santos

**Affiliations:** 1 Brasil Instituto de Ciências Biológicas - Universidade de Pernambuco (UPE), Recife, PE – Brasil; 2 Brasil Pronto Socorro Cardiológico de Pernambuco (PROCAPE-UPE), Recife, PE – Brasil; 3 Brasil Faculdade de Ciências Médicas - Universidade de Pernambuco (FCM-UPPE), Recife, PE – Brasil

**Keywords:** Myocardial Infarction/mortality/mortality, Anisocytosis, Red Cell Distribution Width, Plaque, Atherosclerosis/physiopathology, Prognosis

## Abstract

**Background:**

Acute myocardial infarction is a major cause of mortality worldwide, and atherosclerotic plaque formation is the main pathophysiological mechanism, which results in chronic inflammation that induces erythrocyte maturation and may cause an increase in the red cell distribution width (RDW) index.

**Objective:**

Evaluate the role of the anisocytosis index in patients with acute myocardial infarction in both types of infarctions as a predictor of severity.

**Methods:**

Patients were included in the study according to the inclusion/exclusion criteria, following the hospital routine based on their clinical and laboratory history. Statistical analyzes were performed according to each variable. All conclusions were drawn considering the significance level of 5%.

**Results:**

During the follow-up period, in the 349 patients analyzed, the mortality rate was associated with the variables RDW (CV) and RDW (SD), in those patients who died, an increase was noted, as demonstrated in the multivariate model, for the effects of an acute ST elevation myocardial infarction and the RDW, adjusted for confounding factors (p-value = 0.03 and 0.04). In contrast, the total number of erythrocytes (p-value = 0.00) and hemoglobin (p-value = 0.03) showed a decrease during severe patients’ hospitalization.

**Conclusion:**

The anisocytosis index was a predictive factor of mortality and can be used as an indicator of worse prognosis in patients with acute myocardial infarction.

**Central Illustration:** : Hematological Changes during Seven Days of Hospitalization in Patients with Acute Myocardial Infarction

## Introduction

Acute myocardial infarction (AMI) represents a major health problem worldwide, causing morbidity and mortality.^1^The first symptoms of AMI are manifested during the first hours, and without medical assistance often result in mortality.^2^Infarction may be divided into six categories: infarction due to coronary atherothrombosis (type 1); infarction due to myocardial supply-demand mismatch, not due to coronary atherothrombosis (type 2); infarction leading to sudden death with no opportunity for biochemical or electrocardiographic proof (type 3), infarction related with percutaneous coronary intervention (coronary angioplasty) (type 4a); infarction related to coronary stent thrombosis (4b); and infarction related to myocardial revascularization surgery (type 5). This is a multifactorial disease, which in many cases, makes diagnosis and treatment difficult, hence the importance of using biomarkers that provide information regarding the event severity.^3 , 4^Red blood cell distribution width (RDW), a biomarker also called the anisocytosis index, is expressed by quantifying the size variability of the red blood cells and together with the hematological changes of the erythrocytes, hemoglobin, and hematocrit, are routinely analyzed in cardiac emergencies.^5^The RDW may be reported, depending on the statistical analysis, either as the coefficient of variation (RDW-CV) and/or as a standard deviation, (RDW-SD).

Studies have demonstrated a predictive ability to increase RDW, reflecting several complications during and after the occurrence of infarction.^6 , 7^Regarding chronic inflammation, multifactorial aspects such as age, sex, genetics, hormones, drugs, and diet can modulate the biology and physiology of erythrocytes. Thus these aspects may be considered in the pathogenesis of the infarction where the release of cascade pathways, such as certain cytokines for example, may affect bone marrow regulation with the consequent maturation of the erythrocytes, which affects the rate of erythropoiesis and the size of the circulating erythrocytes, which thereby influence an increase in the RDW.^8 , 9^Atherosclerotic plaque formation, the main cause of infarction, is the result of chronic inflammation that induces the erythrocytes to mature, reflecting an increase in the RDW index.^10 - 12^It is therefore necessary to study and monitor biomarkers in order to assist in clinical results, and thus determine a better immunological and hematological prognosis associated with multifactorial diseases.^13^There are certain advantages to using hematological changes as biomarkers, both are simple and cheap forms of measurements, may be routinely assessed, and can assist in patient stratification in the clinical practice of cardiovascular diseases, they also are especially advantageous in relation to mortality in patients with acute myocardial infarction. Thus, this article aims to assess the changes in the RDW and compare it with the prognosis of patients with acute myocardial infarction during seven days of hospitalization in an emergency hospital.

## Methods

### Study design

This was a prospective, observational study, with data collected from January to September 2018, with infarcted patients, at a university hospital, which is a reference for cardiology. Patients were selected and divided into two groups, according to the type of infarction: those with ST elevation (STEMI), and those without ST elevation (non-STEMI). The clinical, electrocardiographic, and laboratory assessments were performed by the doctor on duty and reviewed by the researchers. The monitoring and treatment of patients followed the institutional protocols. General information such as age, gender, presence of comorbidities, and clinical outcomes, were obtained from the patients’ medical records. The patients were followed up for seven-day period and the outcomes were recorded according to the exact information in the medical records, by the attending medical team.

### Study population

Patients admitted to hospital with AMI during the study period were included. The study excluded patients younger than 18 years, pregnant women, patients with hematological or oncological diseases, previous use of corticosteroids or chemotherapy, and those readmitted after hospital discharge. All patients signed an informed consent form to participate in the study.

### Definition of terms and study variables

Laboratory tests were performed with blood samples collected daily by venipuncture until 9 a.m. Strict quality control criteria were adhered to. Laboratory analyzes were performed with the Sysmex XE-2100 system (Sysmex Europe GmbH, Norderstedt, Germany). The cut-off points for the hematological variables analyzed were Hematocrit (%): Women (35 - 47), Men (40 - 54); Red blood cells (millions / mm ^3^ ): Women (4.0 - 5.6), Men (4.5 - 6.5); Hemoglobin (g / 100 ml): Women (12 - 16.5), Men (13.5 - 18); Platelets: 140,000 to 450,000 (µl) for both sexes; RDW-CV 12 at 14.4%; and RDW-SD 38.6 to 49.1fL, for both sexes.

Potential risk factors associated with AMI such as demographic characteristics (age, gender), systemic arterial hypertension (blood pressure ≥ 140 x 90 mmHg), diabetes mellitus (plasma glucose above 126 mg/dL), sedentary lifestyle (regular practice of physical exercise or not), were adjusted for the statistical model.

During the first twenty-four hours of admission, patients were also classified as infected (sepsis) and uninfected, following the systemic inflammatory response syndrome criteria, plus a documented or presumed infectious focus (use of antibiotics).

### Statistical analysis

The categorical variables were presented through absolute and relative frequencies and compared through the Chi-Square test (χ ^2^ test). Quantitative data with normal distribution were presented through mean and standard deviation and comparisons between groups were carried out using the unpaired t-Student test, in the comparison between characteristics of patients with acute myocardial infarction related to death and related to ST elevation and non-ST elevation. The test applied to prove normal distribution was the Kolmogorov-Smirnov test. A GGE (Generalized Estimating Equations) for repeated measure model was applied to estimate the effect over time of each laboratory measure related to death during hospitalization. A multivariate model of the effect of ST-Elevation and RDW on mortality, adjusted for confounding factors, was undertaken. The level of statistical significance adopted was 5%. After transferring the data compiled in Word and Excel to the STATA program, the entire database underwent a three-point verification process to detect possible inconsistencies and typos before constructing statistical reports.

### Ethics statement

This study is part of the project line (Biomarkers in Clinical Research) approved by the Ethics Committee of the Hospital Complex HUOC/PROCAPE at the Universidade de Pernambuco, under CAAE: 51802115.7.0000.5192 (Brazil Platform). All the procedures involved in this study are in accordance with the Declaration of Helsinki of 1975, updated in 2013. The Informed consent was obtained from all participants included in the study.

## Results

There were changes in the hematological data of infarcted patients during the seven days of hospitalization, with an increase in the RDW according to the length of hospital stay, both for those patients who died and for those who did not, with no association with the type of heart attack ( Central Illustration ). The levels of erythrocytes, hemoglobin, and hematocrit decreased throughout the hospitalization period, regardless of the type of infarction ( Figure 1 ).

**Figure 1:** – Laboratory measures of patients who died (dashed line) and who did not (straight line) during the 7 days of hospitalization according to the in-hospital death condition.

A total of 349 research participants were included in this study. There was a predominance of acute ST elevation myocardial infarction (STEMI) (70.4%). Table 1 presents the characteristics of patients with STEMI and non-STEMI. Adding levels to the general table, the RDW levels, both the RDW (CV) and the RDW(SD) analyzed during the first seven days of hospitalization, presented a very similar level between the two types of AMI.

**Table 1:** – Characteristics of patients with acute myocardial infarction related to ST elevation and non-ST elevation

Features	Total (n = 349)	Non-ST (n = 103)	ST (n = 246)	p-value
**Age (Mean ± SD)**	64.4 ± 12.7	67.9 ± 13.36	63.2 ± 12.1	0.008
**Sex: Male**	222 (63.6%)	47 (45.6%)	80 (32.5%)	0.020
**Risk factors**				
**SAH**				
Yes	254 (72.7%)	88 (85.4%)	166 (67.4%)	0.001
No	95 (27.3%)	15 (14.5%)	80 (32.5%)	
**DM**				
Yes	128 (36.6%)	39 (37.4%)	89 (36.1%)	0.760
No	221 (63.2%)	64 (62.1%)	157 (63.8%)	
**Dyslipidemia**				
Yes	133 (38.1%)	50 (48.5%)	83 (33.7%)	0.009
No	216 (61.8%)	53 (51.4%)	163 (66.2%)	
**Outcomes**				
**Surgery**				
Yes	22 (6.9%)	13 (12.6%)	11 (4.4%)	0.006
No	325 (96.1%)	90 (87.3%)	235 (95.5%)	
**Angioplasty**				
Yes	174 (49.8%)	22 (21.3%)	152 (61.7%)	0.000
No	175 (50.14%)	81 (78.6%)	94 (38.2%)	
**Death**				
No	305 (87.3%)	94 (91.6%)	211 (85.7%)	0.159
Yes	44 (12.61%)	9 (8.74%)	35 (14.23%)	
* RDW % (CV)	13.8 (± 2.25)	13.5 (± 0.3)	13.2 (± 1.3)	0.817
* RDW fL (SD)	44.1 (± 10.2)	43.3 (± 4.3)	43.5 (± 4.7)	
**Sepsis**				
Yes	24 (6.8%)	8 (7.7%)	16 (6.5%)	0.671
No	325 (93.1%)	95 (92.2%)	130 (93.5%)	

SAH: systemic arterial hypertension; DM: Diabetes mellitus; RDW: red cell distribution width*mean and standard deviation of the RDW (coefficient variation) and RDW (standard deviation) obtained during the first seven days of hospitalization. ST: segment elevation myocardial infarction.

Significant differences were found in the variables age, occurrence of systemic hypertension artery, sedentary lifestyle, surgical procedures, and angioplasty, as described in Table 1 . The statistical analysis was performed comparing the general characteristics to the mortality of patients ( Table 2 ). There was a mortality rate of 44 (12.6%). Most of those who died were male patients with ST elevation (14.23%), a mean age of 63 years. Hospital discharge presented in Table 1 was the most prevalent outcome (87.3%) and was predominant in patients with no ST elevation (91.6%).

**Table 2:** – Characteristics of patients with acute myocardial infarction related to death

Characteristics	No death (n = 305)	Death (n = 44)	p-value
**Age (Mean ± SD)**	63.5 ± 12.7	72 ± 10.1	0.008
**Sex: Male**	194 (63.6%)	28 (63.6%)	0.997
**Comorbidities**			
**SAH**			
Yes	217 (71.1%)	37 (84.0%)	0.070
No	88 (28.8%)	7 (15.9%)	
**DM**			
Yes	107 (35.0%)	21 (47.7%)	0.104
No	198 (64.9%)	23(52.2%)	
**Dyslipidemia**			
Yes	118 (38.6%)	15 (34.0%)	0.557
No	187 (61.3%)	29 (65.9%)	
**Outcome**			
**Surgery**			
Yes	22 (7.2%)	2 (4.6%)	0.513
No	283 (92.7%)	42 (95.4%)	
**Angioplasty**			
Yes	152 (49.8%)	22 (50.0%)	0.984
No	153 (50.2%)	22 (50.0%)	
**Sepsis**			
Yes	12 (3.9%)	12 (27.2%)	0.000
No	293 (96.0%)	32 (72.7%)	

SAH: systemic arterial hypertension; DM: Diabetes mellitus; RDW: red cell distribution

Laboratory data was obtained over the seven days through the mean difference in laboratory measurements among patients with ST elevation ( Table 3 ). There was a decrease in hematocrit and hemoglobin levels of erythrocytes. The total erythrocyte count presented a statistically significant difference in this assessment.

**Table 3:** – Estimation of laboratory data over time and mean difference in laboratory measures among patients with ST-segment elevation. Multivariate logistic regression model and p-value

Laboratory measures	Effect over time	ST evaluation	p-value	ST evaluation adjusted	CI (95%)	p-value
Erythrocytes^a^	-0.078	-0.022	0.751	-0.143	-0.28 to -0.01	0.041
Hemoglobin^a^	-0.280	0.092	0.679	-0.369	-0.81 to 0.08	0.107
Hematocrit^a^	-0.629	0.266	0.644	-0.762	-2.06 to 0.61	0.287
Platelets^a^	-0.110	6.57	0.404	5.66	-11.1 to 22.4	0.507
RDW (SD)^a^	0.464	-0.127	0.879	0.005	-0.03 to 0.04	0.756
RDW (CV)^a^	0.080	-0.302	0.102	-0.025	-0.06 to 0.07	0.123

RDW: red cell distribution width.^a^Adjusted for sex, age, hypertension, dyslipidemia, surgery, and angioplasty.

The level of the total erythrocyte count varied to -0.078, with a decrease in the levels, consequently causing a negative effect, according to the type of infarction, over time. When adjusted for the factors: sex, age, hypertension, sedentary lifestyle, surgery, and angioplasty, anisocytosis had a positive effect over time in patients with and without ST elevation. A statistical significance was observed for RDW (CV) and RDW (SD), as well as for the total erythrocyte count and hemoglobin concentration, which were (p < 0.006) and (p < 0.032) respectively ( Table 4 ).

**Table 4:** – Estimation of laboratory data over time and mean difference of laboratory measures over time among patients who died and did not die during hospitalization. Multivariate logistic regression model and p-value

Laboratory measures	Effect over time	ST elevation	p-value	ST elevation adjusted	IC (95%)	p-value
Erythrocyte^a^	-0.077	-0.362	< 0.001	-0.243	-0.42 to -0.07	0.00
Hemoglobin^a^	-0.283	-0.922	0.002	-0.619	-1.18 to -0.05	0.03
Hematocrit^a^	-0.620	-2.56	0.001	-0.730	-2.30 to 0.84	0.36
Platelets^a^	-0.09	-7.040	0.404	-7.160	-28.8 to 14.5	0.51
RDW (SD)^a^	0.431	2.619	0.013	2.277	0.14 to 4.41	0.03
RDW (CV)^a^	0.073	0.76	0.001	0.531	0.02 to 1.04	0.04

RDW: red cell distribution width.^a^Adjusted for age, hypertension, and septicemia.

## Discussion

The data in this study has assessed changes in the anisocytosis index (RDW) in patients with AMI in the two types of infarctions, those with STEMI and non-STEMI. The hematological indexes routinely measured in the emergency laboratory exams assessed in this study demonstrated that the presence of anisocytosis in infarcted patients is associated with higher mortality in acute myocardial infarction, especially in those with ST elevation (STEMI). Complete blood count data are routine in cardiac emergencies and can contribute to the medical care provided by using readily accessible information.

During the last decade, there has been a growing interest in studies that have focused on the use of RDW as a new marker for the prognosis and severity of cardiovascular diseases.^8 , 10 , 12^Ye et al.,^14^analyzed the usefulness of RDW in patients with peripheral arterial disease and reported a positive association between increased RDW and mortality.^14^This study described the patients’ conditions, on admission and over the seven days of hospitalization, according to routine hospital follow-up, including the evaluation of risk scores used by the cardiology team. Most of the population assessed was made up of older people, with a mean age of 64 years ( Table 1 ), a condition that is associated with cardiovascular diseases and the severity of the disease.^15 - 17^

Our findings are in line with the study by Arbel et al.,^18^which reported that in patients with ST elevation, the anisocytosis index was associated with higher mortality rates and maintained an influence over the effects of a pathophysiological sedentary lifestyle and, consequently, the diagnosis and treatment of patients with AMI. When comparing the RDW levels in patients both with ST elevation and without, no statistical difference was observed between the two conditions. However, a progressive increase was observed concerning the reference levels.

The study by Vaya et al.,^19^with 199 infarcted patients, concluded that RDW (CV) levels above 14% were directly associated with a sixfold increase in cardiovascular events, even when adjusted for anemia. Water intake may predispose individuals to a greater future risk for adverse cardiovascular events, since there is evidence that acute hypohydration impairs vascular function and blood pressure regulation,^20^and in this study the electrolytic concentrations in the patients were controlled.

In general terms, the multivariate analysis presented an increase in anisocytosis, when compared to the evolution during the seven days of hospitalization among patients who died and did not die. Determining the RDW may be able to identify rheological changes in the properties of red blood cells, influencing, for example, the aggregation of cells and consequently the viscosity and the rate of blood flow, generating adverse consequences, depending on the patient.^21^

High levels of anisocytosis indicate the production of immature cells by the bone marrow, thereby affecting its activity.^22^An increase in blood viscosity is related to changes in the anisocytosis index as an effect of the increase in the cell aggregation and fragmentation process, as reported by Neuman et al., who identified a significant difference in the survival of patients with unstable angina who had high levels of red blood cell aggregation during hospitalization. This demonstrates that the anisocytosis index may be related to the blood flow rate and micro-occlusion capacity, directly influencing patients with acute myocardial infarction.^23 - 25^

The present study has demonstrated that the RDW is statistically significant when analyzed together with hematological measurements (erythrocytes, hemoglobin, hematocrit, and platelets) over time, when using the regression calculation that considers the mortality variable.

The multivariate analysis presented a pattern of decreased hematological variables in contrast to the progressive increase in the RDW-CV and RDW-SD, according to the days of hospitalization. This finding may have occurred because an alteration in the erythrocyte pattern in hypoxic conditions directly alters essential functions, and increases the inflammatory process and oxidative stress. In addition, it may interfere in the formation of many complexes, which are crucial for homeostasis as a whole, affecting erythropoiesis, which induces deficient erythrocyte maturation, partially in the RDW.^26 - 28^

The patients assessed in this study presented with hemoglobin levels below the normal range. In cases of anemia, a change in the RDW is due to insufficient erythrocyte maturation, in an attempt to supply the oxygen supply, since the presence of anisocytosis is a heterogeneous population of erythrocytes, caused by disorders in the hemoglobinization phase.^29^This reduction is explained as a consequence of obstructions in the coronary artery, which consists of a loss in blood supply, and results in ischemia and cell death throughout the region supplied by the artery.^30^However, this depends on the severity and duration of flow deprivation.^31^

An inverse association of anisocytosis with hemoglobin levels was observed. These data are related to the fact that a decrease in hemoglobin is an indicator of low oxygenation due to pathologies such as infarction, which may lead to several adverse hematological variables, for example, an increase in RDW.^32^

Coronary angioplasty procedures were performed in 49.8% of the patients. However, this procedure was considered to be more necessary in patients with ST-elevation (p < 0.000). A decrease in the post-angioplasty flow has previously been associated with an increase in RDW levels, and is always related to a poorer prognosis.^33^

Angioplasties, as well as other surgical procedures, also directly affect the patient’s profile, as well as the tissue factor in the role of the disease, making the patient immunologically more fragile, since the history of septicemia is directly associated with the highest number of deaths and these factors are intrinsically related.^34^

The present study is limited because the patients were selected in a single center; therefore, the results can reflect the local practice. To lessen this limitation, we increased the sample size, used standardized and predetermined protocol to minimize possible bias. The information about comorbidities and treatment conditions was extracted from medical records, and within the routine of the cardiac emergency room, which may have potential biases. Another important limitation is that the study did not allow adjusting the results for other indicators of severity, such as renal failure, use of anticoagulants, and bleeding. These variables are being the object of further research by the group.

## Conclusions

The role of the anisocytosis index in patients with AMI in both types of infarctions was analyzed showing a predictive factor of severity during seven days of hospitalization. The study has sought to provide a better understanding of the profile of the RDW index, together with the hematological profile for predicting outcomes in infarcted patients.
